# Simple Chemical and Cholinesterase Methods for the Detection of Nerve Agents Using Optical Evaluation

**DOI:** 10.3390/bios13120995

**Published:** 2023-11-22

**Authors:** Aneta Břízová, Vladimír Pitschmann

**Affiliations:** 1 Faculty of Biomedical Engineering, Czech Technical University in Prague, Nám. Sítná 3105, 272 01 Kladno, Czech Republic; pitschmann@oritest.cz; 2 Oritest Spol. s r.o., Čerčanská 640/30, 140 00 Prague, Czech Republic

**Keywords:** nerve agents, chemosensors, biosensors, colour reactions, fluorescence, cholinesterase reaction

## Abstract

The extreme toxicity of nerve agents and the broad spectrum of their physical and chemical properties, enabling the use of these agents in a variety of tactical situations, is a continuing challenge in maintaining the knowledge and capability to detect them, as well as in finding new effective methods. Despite significant advances in the instrumentation of the analysis of nerve agents, relatively simple methods based on the evaluation of colour signals (absorption and fluorescence), in particular those using the cholinesterase reaction, continue to be of importance. This review provides a brief presentation of the current status of these simple methods, with an emphasis on military applications, and illustrates the high interest of the professional community in their further development. At the same time, it also contains some peculiarities (high reliability and durability, resistance to extreme climatic conditions, work in deployed means of protection, low purchase prices, economic availability especially in a state of war, etc.) that the authors believe research and development of simple methods and means for the detection of nerve agents should respect.

## 1. Introduction

Recently, in the context of the escalated international political situation in the world, increased attention has been paid to weapons of mass destruction, including chemical weapons, and protection against them. Basic counter-chemical measures include the use of individual and collective protection, decontamination (detoxification) and detection. Detection in its full spectrum, i.e., detecting the presence of chemical-warfare agents (CWAs), determining their concentration or quantity, and identifying them, appears to be the primary and probably the most technically challenging. The problem of detection is complex and must be addressed in both public protection and armed forces conditions.

In the history of chemical weapons, the modern era of which began as early as World War I, a variety of CWAs with diverse physical and chemical properties and toxic effects has emerged, placing new and more demanding requirements on detection. In the early 1990s, with the adoption of the Chemical Weapons Convention (Paris, 1993), chemical-weapons development still seemed to have reached its peak. However, advances in chemical technology (e.g., chemical microtechnology), combinatorial chemistry, protein engineering, peptide bioregulator synthesis, nanotechnology, or micro/nanoencapsulation suggest that, despite the efforts and unquestionable successes in chemical-weapons control to date, these warfare means may yet experience a “renaissance” of certain sort [1].

Currently, the detection of nerve agents (NA), which are considered to be the “ideal” CWA, is of particular interest to the scientific community. However, a glance at the literature (mainly professional and scientific journals) shows that a disproportionately larger part of the research is carried out in civilian (public and private) universities and research institutes, often with no apparent link to the defence or security sector; for example, in the 1950s–1960s, the opposite was true.

The top research and development institutes usually prefer modern analysis systems used in laboratory expertise or in securing critical infrastructure elements (e.g., underground railways), in the military, especially in central military laboratories. These modern technical means can provide a large amount of information and often previously unimaginably low detection limits. However, for the protection of the population, and especially in the military, relatively simple detection devices, including personal detectors, have traditionally been important and widely adopted as a means of survival in extreme combat conditions, when separated from troops, or in other difficult-to-predict situations. Traditional and simple indicators offer a cheap, easy, and quick way to perform NA screening, which can be supplemented with additional instrumental analysis if necessary [2,3].

This paper deals with simple chemical and biochemical methods (means) of NA detection with optical (colourimetric or fluorometric) evaluation. Due to the specific effect of NAs and their extreme toxicity, the main focus is on cholinesterase methods. The work is mainly addressed to military specialists and security experts. Some of the findings and conclusions are based on our own experience during the long-term research and development of methods and means for the detection of CWA/NA, a fact that influenced the choice of references to a significant degree. The aim of this work is to draw chemists’ attention not only to physicochemical instrumentation of analytical control, as is the prevailing trend today, but also to classical analysis methods.

## 2. Characteristics of NAs

### 2.1. The Mechanism of Action

NAs are biologically active organophosphorus compounds that share a common mechanism of action based on the inhibition of hydrolases, primarily acetylcholinesterase (AChE). Inactivation of this enzyme leads to the accumulation of the neurotransmitter acetylcholine in the cholinergic nervous system, resulting in endogenous poisoning of the body. In addition to AChE, other enzymes are also inhibited, namely butyrylcholinesterase (BChE), also known as pseudocholinesterase, as well as breast-milk lipase, renal phosphatase, amylase, and others. AChE is involved in the transmission of nerve impulses in many parts of the body. However, it plays a crucial role in neuromuscular junctions and within cholinergic synapses, where it acts as a catalyst for the hydrolysis of acetylcholine.

When a nerve impulse occurs, acetylcholine is released from the reservoir and, by binding to specific receptors on the end plate, alters the electrical potential of the excitable cell, changing its permeability to certain ions and, thus, allowing the transmission of the nerve impulse. The duration of action of acetylcholine must be short to allow the excited cell membranes to regenerate rapidly and receive new signals. The task of inactivating acetylcholine and rapidly removing it from the system is performed by AChE, which catalyses the hydrolysis of acetylcholine (by acetylation with serine) to form choline and acetic acid. Under optimal conditions, each molecule of the enzyme hydrolyses about 15,000 molecules of acetylcholine per second. The action of organophosphorus NAs is that they covalently bind to the enzyme at the active site via the serine (OH group), phosphorylate it, and irreversibly inhibit it. The consequence of this inhibition is continuous signal transduction and overstimulation of the corresponding cells [4,5].

### 2.2. Classification

The first group of militarily important organophosphorus NAs consists of compounds developed in Germany shortly before and during World War II, later called G-series or second-generation CWAs. The very first substance of this type, synthesised in 1936 as part of a programme to develop new insecticides by a team of chemists led by Gerhard Schrader, was tabun (GA). Two years later, Schrader’s team prepared sarin (GB), and, in 1944, Richard Kuhn (Nobel Laureate, 1938) prepared the even more toxic soman (GD). During World War II, Germany built factories to produce these substances and fill them into artillery and aerial munitions, producing at least 12,000 tons of tabun, 60 tons of sarin, and 3 tons of soman [6]. After World War II, in the 1950s, the U.S. produced more than 16,000 tons of sarin and the Soviet Union (in the 1960s) produced more than 7000 tons of sarin and 9000 tons of soman. Just for comparison, in the 1980s, at the time of the Iraq–Iran War, Iraq produced about 210 tons of tabun and 800 tons of sarin (more precisely, its mixture with cyclosarin).

The second militarily important group of organophosphorus NAs is the V series of agents (so-called third-generation CWAs), derived from compounds studied in the 1950s in Sweden, the UK, and Germany originally for agricultural or medical purposes; their prototype is the insecticide amiton (VG), prepared in 1952 by the British chemist Ranajit Ghosh. Most of the interest of military planners has focused on VX (the USA produced about 4500 tonnes of VX in the 1960s) and its analogue R-33 (the Soviet Union produced about 15,000 tonnes of R-33 in the 1970s). While the G-series substances (especially GB) can be considered highly effective inhalation poisons, the less volatile V-series substances are particularly notable for their extremely high percutaneous toxicity. Selected data on the physiological activity of G- and V-series substances are presented in Table 1.

During the research and development of new organophosphorus NAs, hybrid compounds, the so-called GV series has been prepared, the chemical structure of which is a combination of the two previous groups of G and V substances. These compounds are believed to have been studied in the 1970s as part of the US IVA program, which was the development of binary chemical munitions. The aim of this programme was probably to bridge the relatively large differences in volatility between G and V, thus allowing their more versatile use in the field. In the literature, GP is cited as a typical representative [7,8].

Recently, the group of organophosphorus agents of the “Novichok” type as so-called fourth-generation CWAs, sometimes also referred to as A-series agents, has been widely discussed by experts and the general public. It is believed that these compounds, also of a hybrid nature, were studied in the 1980s–1990s in the Soviet Union, probably in the form of binary formulas (it even seems that the name “Novichok” originally referred rather to single precursors). The most well-known representatives of this group are the substances A-230, A-232, and A-234, whose structural formulae are known in two forms, one of which is also newly listed in List 1 of the Chemical Weapons Convention [7,8]. The potential warfighting properties of these agents will be severely limited by their extremely low volatility; methods of conversion to a warfighting state allowing percutaneous impact are apparently under consideration.

**Figure 1:**
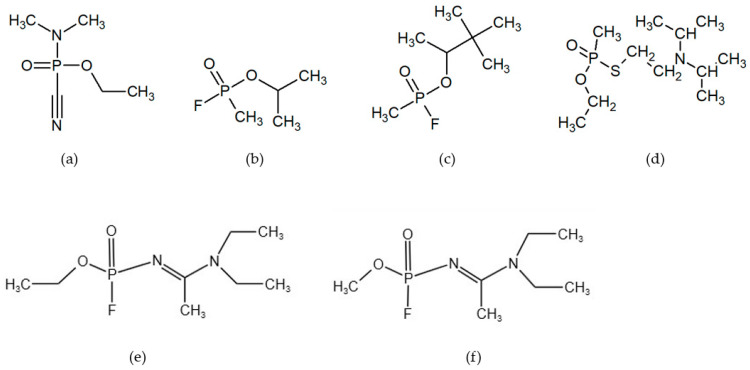
Selected nerve agents: (**a**) tabun GA, (**b**) sarin GB, (**c**) soman GD, (**d**) VX, (**e**) A-232, and (**f**) A-234.

An overview of the chemical formulae of selected nerve agents is shown in Figure 1.

## 3. Traditional Chemical Methods

### 3.1. Reactions of Hydrolytic Products

The German chemist R. B. R. Schoenemann presented a method for detecting GA during World War II by using its hydrolysis to form CN ions, which, with Cu(II), provide oxygen in statu nascendi (in the state of birth). This atomic oxygen then oxidized luminol under the influence of chemiluminescence. In analytical practice (but mainly for the detection of HCN in the air), various aromatic amines whose oxidized forms are intensely coloured have been used. Later, GA was also detected by other methods of cyanide analysis, in military practice, for example, by the Koenig–Zincke reaction (with conversion to cyanogen chloride) to form polymethine dyes [9] or by benzoin condensation catalysed by cyanide [10]. Based on the benzoin condensation principle, a detection tube containing a layer of silica gel saturated with KNaCO_3_ and a vial containing a solution of *p*-nitrobenzaldehyde in pyridine was used in the former Czechoslovak army; a red–violet salt of dinitrobenzoin was formed. The current range of colourimetric, as well as fluorescence, methods of cyanide detection is varied and wide [11,12,13], but their suitability for the analysis of GA and its differentiation from other potential NAs (e.g., GB, GD, GP, VX, and A-230/4) remains to be verified.

All organophosphorus fluoridates can be broken down by hydrolysis to fluorides, which are evidenced by colour reactions, in military practice mainly by decolourization of some heavy metal complexes such as alizarin/Zr(IV) [14,15].

The isopropyl group (GB) reacts with concentrated sulphuric acid to give isopropyl sulphuric acid, which condenses with *p*-dimethylaminobenzaldehyde to form a polymethine red dye. Similarly, the pinacolyl group (GD) reacts with sulfuric acid and vanillin to give a red–violet dye [16]. The reactions are quite challenging to perform but are suitable for field chemistry laboratories.

The presence of 2-dialkylaminoethanethiols as hydrolytic products distinguishes the V-series compounds (VX, R-33, and others) from all other types of organophosphorus CWAs. In military practice, the ability of these hydrolytic products to reduce and thereby decolourize certain triphenylmethane dyes such as Malachite Green or Guinea Green [17], to reduce Ellman’s reagent to give a yellow-coloured product [18,19], Folin–Ciocalteu’s reagent [20] to give a green product, or 4-chloro-7-nitrobenzofurazan [21] to give similarly coloured compounds, has been exploited. Also of interest is the ability to detect thiol hydrolysis products using a specific fluorogenic reagent based on fluorescein [22].

It is understandable that individual reactions of hydrolytic products are not selective; a relatively large number of different agents react similarly. However, the frontal use of several reactions side by side can already, with a big probability, confirm or exclude the presence of a certain NA in the sample.

### 3.2. Schoenemann Reaction

**Figure 2:**
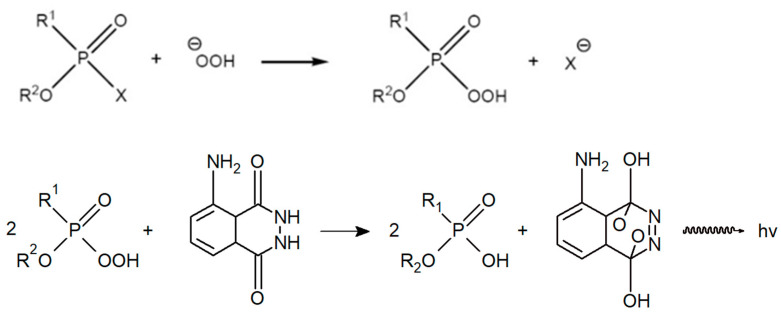
Schoenemann reaction with luminol.

The aforementioned R. B. R. Schoenemann developed a method to detect all G-series NAs based on their reaction with hydrogen peroxide to form peracids, which, in an alkaline environment, oxidize luminol and some aromatic amines to characteristically coloured products [23]; the reaction scheme is shown in Figure 2. The reaction with hydrogen peroxide, which was described in detail in the 1950s, e.g., in Canadian or Swedish defence laboratories, thus plays a fundamental role [24,25]. The reaction takes place in the presence of hydroxide ions, which react competitively, so it is necessary to ensure an optimum pH; opinions are not uniform, and a fairly wide range of pH 7–10 is given. Historically, three basic modifications of the Schoenemann amino peroxide method have evolved—colourimetric, fluorescence and chemiluminescence [23,26]. The individual modifications differ in the reagents used and the methods of evaluating the reaction product. The colourimetric modification usually employs redox indicators, such as benzidine, diphenylamine, *p*-phenylenediamine or triarylmethane leuco base) [24]; the fluorescence modification is based on the use of indole and the chemiluminescence modification on the use of luminol.

In the past, the Schoenemann method was often applied in detection tubes or simple mobile field laboratories; for example, the M19 (CBR Agent sampling and analysing kit), introduced in 1964, contained *p*-amino-*o*-ethoxy-*o*-sulfo-diphenylamine in tablet form [27]. As for more sophisticated instrumentation, e.g., the US armed forces used the M5 Automatic G-Agent Fixed Installation Alarm based on fluorescence (indole solution, hydrogen peroxide, or sodium perborate) from 1959 [28], which was gradually modified to the M6 (Navy) and M7 (plus E59 under development) types [29]. In the Soviet Union, a similar analyser, the GP-1, was introduced [30]. Also, in former Czechoslovakia, the development of an automatic GB detector based on the principle of fluorescence detection was included in the scientific research plan for 1959 [31]. A brief overview of some of the modifications of the Schoenemann reaction applied in the past is given in Table 2.

The Schoenemann method of detecting organophosphorus CWAs is still of interest today, as evidenced by some new proposals to optimize the reaction, such as a colourimetric method with the extraction of the reaction product into chloroform for application in field laboratories [16], increasing the reaction temperature [32], or the use of a chemiluminescence sensor system MgAl-LDH)-luminol-H2O2 (LDH = layer double hydrolysis) [33].

The Schoenemann method is relatively simple and can be used well in both laboratory and field conditions. The most significant interfering influences are other alkylating or acylating agents (e.g., phosgene) and common oxidants (in practice, e.g., decontaminating agents with active chlorine).

### 3.3. Group Reactions

NAs that contain (cleave off) an amine group (GA, V-series, GV, or Novichok-type substances) react with Dragendorff’s reagent to form an orange to orange–red precipitate. Other group reagents for amines and alkaloids may also be used to detect these substances.

Organophosphorus NAs can also be detected using 4-(*p*-nitrobenzyl)pyridine, a reagent designed to detect alkylating agents [34]. In alkaline environments, a blue colouration is produced with NAs, similar to that with sulphur or nitrogen mustard; yellow-to-red colouration is provided by, for example, phosgene, cyanogen chloride, chloroacetophenone (CN), or 2-chlorobenzylidene malononitrile (CS).

Methods based on their reaction with oximes, preferably aldoximes in the α-position with an oxy or hydroxy group, to form HCN have also been developed in the past in order to detect G-series compounds. For example, a method with monoisonitrosoacetone has been proposed, in which HCN is demonstrated by conversion to a perchlorate and its reaction with a polymethine dye [35]. A variant with isonitrosobenzoylacetate has been applied in an American automatic detector. If diisonitrosoacetone is used, an unidentified red-coloured product is formed in addition to HCN [36]. G-type substances react with hydroxamic acids (usually potassium benzohydroxamate) to phenyl isocyanate, which hydrolyses to aniline in an alkaline medium; aniline can then be demonstrated, for example, by condensation with *p*-dimethylaminobenzaldehyde to an azomethine dye [16].

Group reactions have a similar meaning to reactions of hydrolytic products—the detection of Nas is only indicative, but, when chosen correctly, can provide very useful information.

### 3.4. Other Methods

For the detection of liquid CWAs and their aerosols, simple methods based on the use of simple technical means with a visual assessment of colour changes due to (a) the solubility of organic dyes and change in pH of the medium, (b) the decomposition of compounds to form coloured components, or (c) the chemical reaction with chromogenic reagents are usually used. The organic dyes, compounds, and chromogenic reagents are immobilized on a surface carrier. The first group includes, for example, multicomponent M8 detection strips (F1, PP-3, and CALID-3) to detect and discern mustards, G-series agents, and V-series agents. Detection of G-series substances is provided by the Dye Yellow A2 reagent and the detection of V-series substances by the Dye Green EDA reagent, providing yellow and olive-green colouration, respectively. Detection paper impregnated with the acid–base indicator Bromocresol Green with a yellow–blue colour transition was also used in the past for the detection of liquid VX [16]. An example of the second group of means can be detection paper impregnated with metal coordination complexes, which are decoloured by NAs of the G-series [37]. A third group may include detection paper with a dithiobenzotropone-based reagent [38], which gives a red or blue colour when in contact with liquid samples of the G-series NAs.

An overview of selected traditional reactions used to detect organophosphorus NAs is given in Table 3; “+” means positive, “−” means negative, “?” means unknown.

## 4. Cholinesterase Methods

With the military standardization of V-series agents (VX), the sensitivity of traditional chemical methods was no longer satisfactory; therefore, increased attention was paid to cholinesterase methods. The reduction of enzyme activity after contact with NAs allows for the detection of these cholinesterase inhibitors with high sensitivity since, in this case, one and the same enzyme acts as both a sensitive element and an amplifier of the analytical signal. To illustrate the dynamics of the development of this problem, reference can be made to a review of methods for the determination of cholinesterase inhibition up to about the mid-1950s [39] and to relatively recent reviews that emphasize the design principles of relatively simple cholinesterase-based devices and, in particular, their military applications [40,41].

### 4.1. Enzyme, Substrate, and Acid–Base Indicator

Already during World War II, Richard Kuhn’s team developed a biochemical method to determine AChE activity based on the colour indication of acetic acid released by the hydrolysis of acetylcholine with bromothymol blue (pH 6.0–7.6, yellow–blue)—in the plasma or serum of experimental animals, as a way to evaluate the efficiency of a new generation of CWAs [42]. Based on this method, a rapid test for cholinesterase activity in human blood [43], as well as field methods for the detection of organophosphorus inhibitors, were later developed. In 1964, a simple field detector containing, in addition to the enzyme, the substrate acetylcholine and the acid–base indicators Bromothymol Blue or Phenol Red were described [44]. During further studies, Phenol Red (pH 6.8–8.8, yellow–red) proved to be more suitable. For example, in about 1965, the Soviet GSP-11 automatic tape analyser was introduced with a textile tape sampling of contaminated air, a BChE solution dispenser, and a butylcholine solution dispenser with Phenol Red. Phenol Red is still used in the detection tubes.

The advantage of a system with acid–base indicators is a distinct and unambiguous colour transition. The disadvantage is the problematic use of quantitative analysis and the interference of acidic and basic agents.

### 4.2. Enzyme, Thio-Substrate, and Redox Indicator

**Figure 3:**
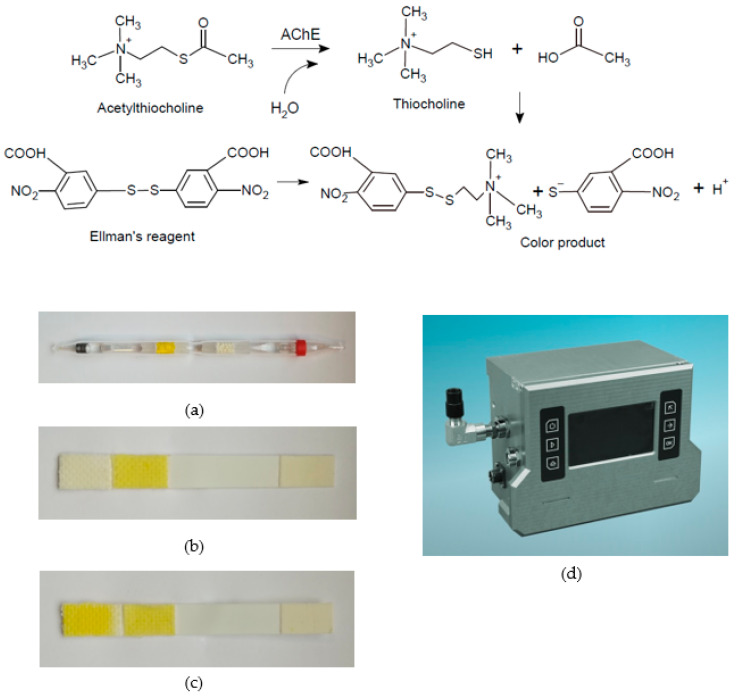
Standard Czech devices work on the principle of cholinesterase reaction using Ellman’s reagent (from above): (**a**) detection tube DT-11; (**b**) personal detector/biosensor DETEHIT positive result; (**c**) personal detector/biosensor DETEHIT blank; (right) (**d**) the newly developed DAPH detection device of nerve agents.

A highly significant modification of the cholinesterase method was the replacement of the specific substrate acetylcholine with the synthetic substrate acetylthiocholine (later also its analogue butyrylthiocholine), which breaks down to create thiocholine, which reduces 5,5′-dithiobis-2-nitrobenzoic acid (DTNB) to a yellow-coloured product [45]. This so-called Ellman’s reagent is perhaps the basis of the most widely used optical methods employed not only in clinical biochemistry and many other fields but also in the military for the detection of organophosphorus NAs [46,47,48]; the basic reaction scheme and examples of use are shown in Figure 3. Some analogues of Ellman’s reagent have also been validated for the detection of NAs, but none of them have yielded improved analytical performance. Other reagents, such as 5-(2-aminoethyl)dithio-2-nitrobenzoate [49], will certainly be the subjects of further study.

Another group of redox indicators suitable for the cholinesterase method is 2,6-dichlorophenolindophenol and its analogues, which are decoloured by reduction. For example, 2,6-dichlorophenolindophenol (blue in the oxidized form) has been proposed for the automated determination of cholinesterase inhibitors for routine clinical laboratories [50] or an area biosensor for the detection of NAs [51]. The analogous N-(2,3-dimethyl-5-oxo-1-phenyl-3-pyrazolin-4-yl)-2-chloro-5-sulfo-4-iminobenzoquinone is also relatively resistant to acidic and basic gases and vapours, but the redox reaction is slower (about 5 min); it has been proposed for a biosensor for NAs detection with a red–white transition [52].

**Figure 4:**
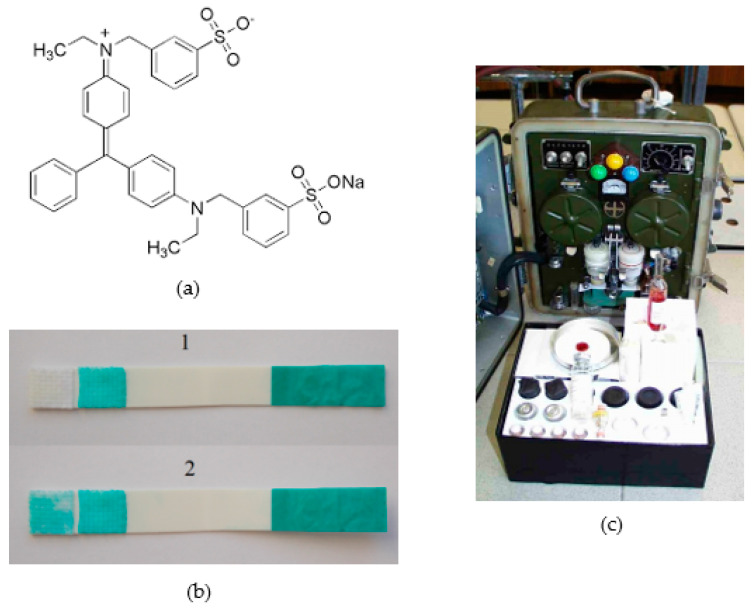
(**a**) Structure of Guinea Green B; (**b**) Guinea Green biosensor for nerve-agents detection: 1—blank, 2—presence of nerve agents. Reprinted from ref. [53]; (**c**) the older Soviet GSA-12 automatic alarm, which works on a similar reaction principle.

Of particular importance in military applications of the cholinesterase method are triphenylmethane dyes, which are decoloured by reduction. The most widely used is probably Guinea Green B, designed for the biosensor for NAs detection [53] and earlier for the Soviet GSP-12 (Figure 4) automatic tape analyser, which replaced the obsolete GSP-11. A mixture of Guinea Green B with another triphenylmethane dye, Fuchsin Basic, which changes the blue colour to red–violet by reduction, was proposed for the preparation of the detection tube and the surface biosensor for NAs detection [54]. In all these means with triphenylmethane dyes, BChE was preferentially used.

The advantage of a system with redox indicators is its possible use for quantitative analysis and a higher resistance to common interferences.

### 4.3. Enzyme and Chromogenic Substrate

An interesting and very successful variant of the cholinesterase method is based on the use of so-called chromogenic substrates, which hydrolyse directly to generate a coloured product; the chromogenic substrate is therefore both a substrate and a chromogenic reagent.

Among the oldest and still used chromogenic substrates are indoxylacetate (3-indolylacetate) and its analogues [55,56,57,58]. Indoxylacetate is colourless; in the absence of a cholinesterase inhibitor, it hydrolyses to a blue–green fluorescent product which is further oxidized to indigo blue. Proposals have been made to accelerate the formation of indigo by the addition of a mixture of potassium ferrocyanide and potassium ferricyanide [59]. A structurally different resorufinbutyrate has been proposed as a fluorogenic substrate along with indoxylacetate [56].

In the 1950s, indophenolacetate with a red-to-blue colour transition was proposed for the measurement of AChE activity [60]. It has recently been described in a colourimetric detector, validated on the pesticide dichlorpyrifos, with an evaluation by smartphone [61]. Even more well known is 2,6-dichlorophenolindophenolacetate, a red-coloured compound which, by reduction (i.e., in the absence of inhibitors), provides the analogous blue 2,6-dichlorophenolindophenol (see redox indicator above). It has appeared, for example, in the design of a detection-tube preparation [62] or in the Canadian Nerve Agent Vapor Detector (NAVD) kit [30] and similar devices. It is still very popular and used in NA detectors, with the drawback of its lesser availability and high cost.

Classical chromogenic substrates include *p*-nitrophenyl acetate or 2-azobenzene-1-naphthylacetate, yellow-coloured compounds that hydrolyse to red products [16]. However, some compounds are known that can be considered as chromogenic substrates only conditionally (they belong rather to the group of nonspecific substrates). A simple field detector of cholinesterase inhibitors, which contains the substrate 6-bromo-2-naphthylacetate and as an indicator a stabilized diazonium salt (Fast Blue B salt), reacting with the resulting 6-bromo-2-naphthol to a distinct azo-coloured dye, is also described in the previously mentioned work [44]. Later, 1-naphthylacetate was proposed as a fluorogenic substrate for pesticide detection [63].

The advantage of using chromogenic substrates is the significant simplification of the analytical system (it contains only two key components); the disadvantage is the lower selectivity.

## 5. Trends and Perspectives

### 5.1. A Few General Premises

In military practice, it is convenient to divide technical devices into several categories, depending on the tasks they will perform, individual detectors, detectors for liquid agents, chemical detectors, automatic detectors and rapid detectors, portable field chemical laboratories, mobile chemical laboratories, and remote detectors [16]. However, it is not yet very common to call some of the technical devices biosensors. However, if these devices use the cholinesterase reaction, they can be considered as biosensors, or at least as devices with a biosensor as a basic functional element (see more in Section 5.3.1). The cholinesterase reaction is applicable in virtually all of these types of devices but with the following limitations: (1) automatic detectors are slower than conventional rapid detectors based on physical principles, e.g., the currently frequently used IMS (ion-mobility spectrometers); and (2) they are not at all suitable for the detection of liquid (or more concentrated) CWAs, where the cholinesterase method is unnecessarily sensitive and costly.

In principle, however, it is not possible to construct a single device that meets all the requirements for chemical reconnaissance and control or field and laboratory analysis. These requirements can only be met by an entire system of technical means, which is also constantly being extended and upgraded. The required content and range of tasks can only be fulfilled by applying the various principles used by the chemical reconnaissance and control equipment, i.e., physical, chemical, biochemical, and physiochemical principles, as well as the different levels of design of the individual equipment. Unilateral preference of any principle or method must sooner or later result in a loss of effectiveness of the whole system. On the contrary, the system must be continuously developed in accordance with the changes taking place in the field of chemical weapons [16].

One of the premises for further development is also the following argument: chromatographic, spectrometric, and other instrumental methods of analysis are commonly used for the laboratory detection of NAs, but these methods do not allow (or allow under difficult conditions) for the in situ detection of NAs. Therefore, various chemosensors with optical signal evaluation and, in particular, highly efficient biosensors, which typically use the enzymes AChE or BChE as biosensitive elements, are desirable in field conditions. In the following text, this problem (especially biosensors) will be addressed in particular.

### 5.2. Development of Chemical Methods

**Figure 5:**
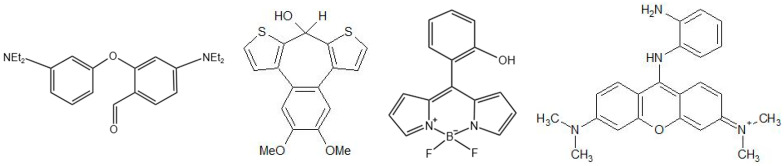
Examples of simpler chemical structures of reagents/chemosensors designed in recent years for the detection of cholinesterase inhibitors.

Research into methods concerning analysing organophosphorus NAs has recently seen a dramatic increase in the design of new simple and rapid colourimetric and fluorometric methods. A large number of reports on new colourimetric and fluorescent indicators or chemosensors, which are perceived as molecular structures used to scan an analyte to produce a detectable change or signal, can be found in scientific journals and other professional sources; the chemical structures of some of these reagents/chemosensors are shown in Figure 5. The functions of these chemosensors are based on hydroxyl activation (mechanism of binding to serine residue), N activation, and the use of metal ions and polymers. In terms of reaction principles, many of them are based on traditional chemical methods. It is not possible to summarize here the multitude of already proposed compounds, therefore we refer to the detailed and available reviews [64,65,66,67,68]. Most of these chemosensors have been tested on simulation agents (mimicry) and, so far, only a small part on real NAs (in this case, military laboratories, emergency services laboratories, etc. have usually been involved in the experiments). It will, therefore, be interesting to find out whether the studies with these mimics have yielded at least partially usable practical knowledge or whether these findings will have to be revised after verification of real CWAs [3].

### 5.3. Development of Cholinesterase Methods

#### 5.3.1. Biosensors

At this point, it is necessary to mention the generally accepted definition of biosensors; they are analytical devices that integrate a biological element (bioreceptor), which recognizes the target molecule (analyte) with a detector (transducer) that converts the analytical signal, which is appropriately registered and evaluated. The biological element can be a tissue, a microorganism, an organelle, a cell receptor, a nucleic acid, or an enzyme, as is the case with the biosensors we are addressing in this article. The analytical signal can be registered by a variety of analytical methods, preferably optical or human senses (naked eye) in the case of field analysis (in situ). Analogously, chemosensors can also be defined (Section 5.2), with the difference that, instead of a bioreceptor, it will contain a chemical reagent.

The current level of technology is very advanced, allowing the construction of biosensors for a variety of purposes, including mobile miniature chips that can be attached to clothing or directly to the skin [69]. It is evident that the development of systems for the registration and evaluation of the analytical signal is very dynamic and promising in many respects (see Section 5.3.4 for more details), but it must be kept in mind that the essence of the cholinesterase method is the properties of the enzymes and their sensitivity to NAs; the sensors and the corresponding design solutions “only“ more or less mediate the analytical signal. These advanced technologies can be very useful in some areas (e.g., medicine, forensic technology, etc.), but, in other areas that require mass dissemination (such as protecting people from the effects of chemical weapons in the field), it may be inefficient or problematic.

#### 5.3.2. Problems with Immobilization and Stabilization of Enzymes

Although AChE and BChE are structurally similar enzymes, their functions in the body differ significantly; the more specific AChE is preferred in analytical chemistry and biochemistry. In general, the principle of most cholinesterase-based biosensors uses AChE [70]. However, available information shows that, especially in military biosensor applications, BChE has been and still is used quite frequently in the past (compared with Section 4); one reason may be better availability. The fundamental difference between the two enzymes is their willingness to catalyse the hydrolysis of esters other than their physiological (specific) substrate. There are also differences in the affinity of the individual inhibitors for the enzymes [16].

In the past, enzymes were often used in solutions (e.g., simple field detectors [44], GSP-11-type analysers and alarms, or some older detection tubes for military use), but, due to efforts to optimize critical parameters, including stability, current biosensor research and development is generally (but not exclusively) focused on enzymes immobilized on carriers. Immobilized enzymes can be defined as enzymes trapped or localized in a specific area of space that retain their catalytic activity and can be used repeatedly or continuously [71]. Methods known from other areas of biotechnology are used to immobilize cholinesterase, most commonly adsorption or covalent bonding to insoluble support, binding to ion exchange, incorporation into gels and foams, cross-linked aggregates, or immobilization by nanostructures, antibodies, and others [72].

For example, the DETEHIT personal detector introduced in the Czech army contains AChE immobilized in the form of a stable enzyme chimaera with the polysaccharide cellulose so that the enzyme remains in the solid phase and its use is polyvalent. A similarly prepared tape with an immobilised enzyme is contained in the DAPH detector, which was developed as part of the Czech project to replace the Soviet GSP-12 analyser (some experimental data in ref. [46]). Innovations in this direction can also be relatively simple, yet with a significant practical impact. For example, microcellulose pellets, traditionally used as enzyme carriers in NA detection tubes, can contain composite and nanocomposite materials with magnesium aluminometasilicate and similar sorbents. In addition to stabilizing the enzymes, this will improve their catalytic activity or detection sensitivity [73]. The enzyme can be used as a commercial product or a laboratory-prepared material, for example, an aqueous suspension of crushed brain tissue (nucleus caudates) of Sus scrofa f. domestica with the addition of stabilizers.

Enzymes used to develop biosensors can also be in the cell structures of microorganisms. This idea is not entirely new either; for example, the AChE activity of various strains of bacteria of the genus *Pseudomonas*, particularly *P. aeruginosa*, was determined some time ago [74,75]. The use of microorganism cells will increase the stability of the enzyme because it is in its natural environment; however, the sensitivity of detection is likely to be lower because the enzyme and NAs will be separated by a larger barrier.

Quite recently, an idea based on so-called nanozyme activity, i.e., the use of nanomaterials that simulate enzymes by their catalytic activity, has appeared in the literature [76,77]. Nanozymes could be used to develop biosensors in combination with or as a substitute for native enzymes. However, so far, only the detection of pesticides is considered. At this point, it is also worth mentioning the so-called mutant or recombinant enzymes prepared by protein-engineering methods [78,79]. The main aim of modification is to increase the stability, sensitivity, and selectivity of the protein (enzyme). For example, the AChE Y408F mutant in *Drosophila melanogaster* was recently identified to have high sensitivity to many organophosphates [80]. In the military, this technique is currently more studied in the field of NA detoxification [81].

The problem of stability also fully concerns substrates and chromogenic reagents. For example, the stability of the substrate acetylthiocholine in water is, according to experience, about 7 days; the stability of butyrylthiocholine is about 30 days. Their stabilities are usually increased by the addition of polar solvents (ethanol or dimethyl sulfoxide) [82], but in modern systems their immobilization on a suitable carrier is preferable. Practical experiences show that the lifetimes of biosensors often depend on the stability of the substrate.

#### 5.3.3. Problems with Selectivity and Sensitivity

All organophosphorus compounds of the NA group, to a certain extent, inhibit AChE or BChE containing a serine amino acid residue in their active centre. Phosphorylation of this residue is the basis for the relatively high selectivity of detection of cholinesterase inhibitors. The result of the detection largely indicates whether the human organism has been or will be affected by NAs as such. However, enzymatic methods are not able to distinguish between the different compounds belonging to NAs, and, therefore, enzymatic methods have to be complemented by other methods, including classical chemical methods. Nevertheless, specific procedures are also known. For example, the differentiation of individual NAs is possible using a colourimetric biosensor (DETEHIT with immobilized AChE is reported), followed by a reaction with nucleophilic reagents (mono- or bipyridinium oxime) commonly used in the treatment of NA poisoning. The differentiation is based on the comparison of spectral data of carrier surface remission and evaluation by artificial neural network methods [83,84].

**Figure 6:**
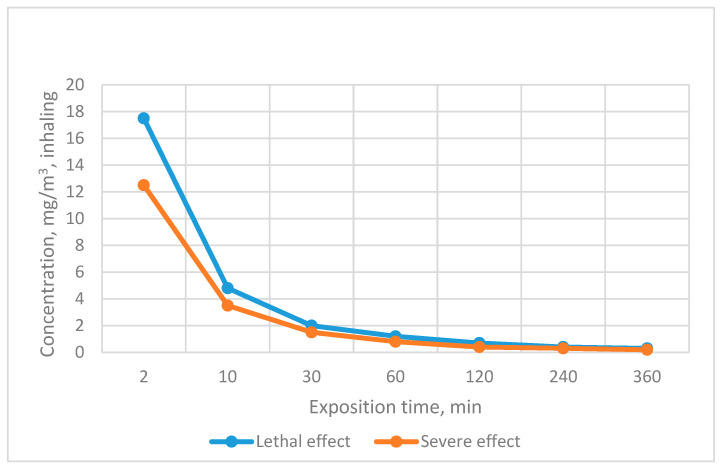
Dependence of GD effect on exposure time.

The detection limit essentially corresponds to the toxic effect of NAs on a living organism. The sensitivity of detection under real conditions can be increased by modifying the reaction system or by improving the registration of the analytical signal. The importance of respecting the necessary detection sensitivity can also be assessed on the basis of the health standards and toxicity data presented in Figure 6 and Table 4 [7,85].

#### 5.3.4. Chromogenic Systems and Evaluations

For chemical methods of NA detection (Section 5.2), research into fluorescence chemosensors with superior sensitivity or dual colourimetric/fluorescence chemosensors has long been preferred. In addition to this enhanced sensitivity, dual chemosensors are also characterized by greater accuracy and reliability; indeed, two independent signals can reduce false positives and false negatives. This trend has also found its way into cholinesterase research. Some fluorogenic substrates, such as the aforementioned indoxylacetate, resorufinbutyrate, or 1-naphthylacetate, have been used in the past to better diagnose and detect cholinesterase inhibitor poisoning. More recently, research has focused on the synthesis of new modern fluorescent sensors with high response rates and good sensitivity, suitable for the construction of test papers and their validation on real NAs [86]. Dual-mode-based optical detection is also beginning to appear in enzymatic biosensors for pesticide detection, where nanomaterials are sometimes used as signal amplifiers or directly as transducers [87]. This approach could also be useful in the development of some types of devices designed for the detection of NAs.

The analyte in contact with the reagents causes a colour change (analytical signal), which can be assessed, e.g., by a smartphone as a possible replacement for conventional colourimeters and spectrophotometers. The advantage of smartphones is that they are widely used in everyday life and various apps and software to convert images into usable data are already available. The method has also been used for the simple determination of neurotoxic substances (the anti-Alzheimer drugs tacrine and galantamine) with AChE immobilized in gelatine gel, evaluated using a smartphone camera [88]. Such a system would be ideal, for example, for the detection of pesticides in food, but, in the military, especially in emergency and combat situations, it would probably be affected by some operational and regime limitations.

And there are two more remarks to be made. It should always and in all circumstances be remembered that the essence of enzymatic methods is the hydrolysis of a suitable substrate, and, therefore, the presence of water in the detection system is necessary. The analysis of a contaminated water sample is without problems from this point of view (water is already contained in the sample), whereas for the analysis of an air sample (when NA is detected on a solid carrier), the water is supplied, for example, in glass ampules or other containers.

Registration of fluorescence can be challenging in the military field as it requires special equipment, even for the simplest optical evaluation, and at least a UV lamp chamber.

## 6. Conclusions

It is hard to ignore the fact that there is a worldwide demand for methods and technical means of detecting NAs and that the scale and intensity of research are responding to this demand. On the other hand, however, the fact that this research does not fully reflect some peculiarities of the detection of NAs in the armed forces or emergency services cannot be overlooked (high reliability and durability, resistance to extreme climatic conditions, work in deployed means of protection, low acquisition cost, economic availability, especially in a state of war, etc.); here, in fact, this detection is reflected in the performance of complex tasks that can be called chemical survey and control. Perhaps only greater awareness is needed. Most research institutes rely mainly on published papers, which are generally not interested in the patent literature, even though this is usually the first source of data on the various methods and means of detecting militarily relevant NAs. There is also the aforementioned overorientation towards the use of simulants in research. Many publications also mention organophosphorus or carbamate pesticides because working with them in the laboratory is similar but much safer than working with NAs. However, this means that, although the reaction principle is the same, the sensitivity of the enzymes to the pesticides is lower, and the verification of real NAs is therefore necessary.

If any new potential CWA with anticholinesterase activity, of a magnitude more toxic than VX, already exists (or will emerge in the near future), then none of the currently established detectors used for military and security purposes, with the exception of those using the cholinesterase biochemical reaction, have the necessary sensitivity and do not provide the necessary level of protection for troops and the population [16]. Just for comparison, if colourimetric chemical methods can detect NA at concentrations of 0.1–10 mg/^3^ in the air or 0.1–10 mg/L in water (fluorometric methods are generally more sensitive), the cholinesterase method is in principle at least 1000 times more sensitive; even the most widely used IMS-based (ion-mobility spectrometry) detectors and analysers cannot compete with it in this respect.

However, the cholinesterase method cannot be the only method of detection of NAs used in practice; it is necessary to respect the diversity of methods in their principle, design, and method of use. Therefore, it is recommended to also accept classical (nonenzymatic) colourimetric methods, suitable as a complement (e.g., confirmation and differentiation of individual NA species), or to address tasks for which cholinesterase methods are unnecessarily complex and expensive (e.g., detection of liquid samples or liquid aerosols). It is also important that the methods (means) developed for the detection of NAs form a coherent whole with those for the detection of other CWAs (vesicants, asphyxiants, bloods, incapacitating, and irritants) or major industrial pollutants (chlorine, sulphur dioxide, sulphane, carbon monoxide, etc.).

## Figures and Tables

**Table 1:** Physiological activity of NAs [7].

CWAs	LCt_50_ (mg min/m^3^) Inhaling	LCt_50_ (mg min/m^3^) Percutaneously	LD_50_ (mg)
GA	70	15,000	1000
GB	35	12,000	1700
GD	35	3000	350
VX	15	150	5

**Table 2:** Comparison of selected modifications of the Schoenemann reaction.

Means/Sample	Method	Indicator	Detection limit (GB)	References
In solution	Colourimetry	o-Dianisidine	0.5 μg/mL	Own results
	Chemiluminescence	Luminol	0.5 μg	[23,26]
	Fluorescence	Indole	0.05 μg	[23,26]
Detection tube (in air)	Colourimetry	o-Dianisidine	0.5 mg/m^3^	Own results
Automatic alarm (in air)	Fluorescence	Indole	<0.01 mg/m^3^	[28]

**Table 3:** A review of some traditional reactions for the detection of organophosphorus NAs; for comparison, the cholinesterase reaction is also given (data based on the authors’ own knowledge).

Reaction	GA	GB/GD/GF	VX/R33	GP	A230/2/4
Schoenemann reaction	+	+	−	+	+
Detection of aminothiols	−	−	+	−	−
Detection of CN^−^	+	−	−	−	−
Detection of F^−^	−	+	+	+	+
Dragendorff’s reagent	?	−	+	+	+
4-(*p*-nitrobenzyl)pyridine	?	+	−	+	?
Diisonitrosoacetone	?	+	−	?	?
Cholinesterase reaction	+	+	+	+	+

**Table 4:** Proposed air detection limits considering health standards (ppm converted to mg/m^3^).

Substance	AEGL-1/2/3 (1 h)	IDLH	Ideal Detection Limit	Necessary Detection Limit
GA	0.0027/0.0332/0.2652	0.099	0.003	0.1
GB	0.0029/0.0344/0.1146	0.115	0.003	0.1
GD	0.0015/0.0149/0.149	0.052	0.002	0.05
VX	0.0002/0.003/0.01	0.003	0.0002	0.003

## Data Availability

No new data were created or analyzed in this study. Data sharing is not applicable to this article.
